# Supplementary material for: Synthesis and Thermo-Responsive Behavior of Poly(N-isopropylacrylamide)-b-Poly(N-vinylisobutyramide) Diblock Copolymer
Source: Polymers (Basel). 2024 Mar 18;16(6):830. doi: 10.3390/polym16060830 (PMC10975249; doi:10.3390/polym16060830)
Supplement: Supplementary file 1 [file polymers-16-00830-s001.zip › polymers-2908676-supplementary.pdf]

Supplementary Materials

Synthesis and Thermo-responsive Behavior of Poly(*N*-isopropylacrylamide)-*b*-Poly(*N*-vinylisobutyramide) Diblock Copolymer

Jun Hyok Yoon[1], Taehyoung Kim[1], and Myungeun Seo[1,2], and Sang Youl Kim[1*]

[1] Department of Chemistry, KAIST, Daejeon 34141, Republic of Korea
[2] KAIST Institute for Nanocentry, KAIST, Daejeon, 34141, Republic of Korea
* Correspondence: kimsy@kaist.ac.kr; Tel.: +82-42-350-2834

Supplementary Figures

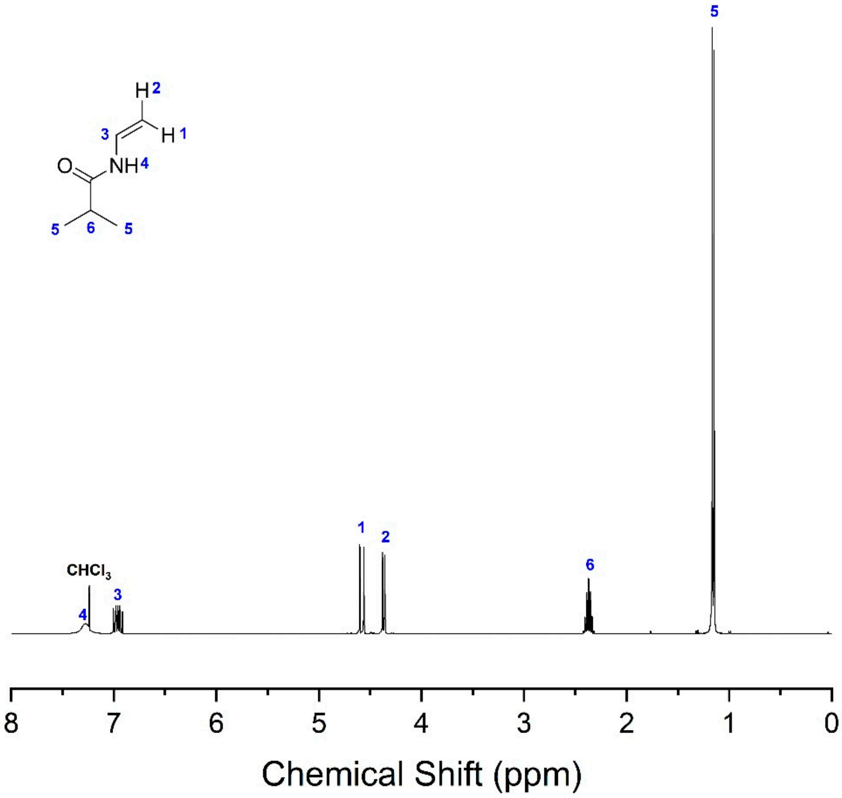

Figure S1. ^{1}H NMR spectrum of synthesized *N*-vinylisobutyramide monomer (400 MHz, $CDCl_3$).

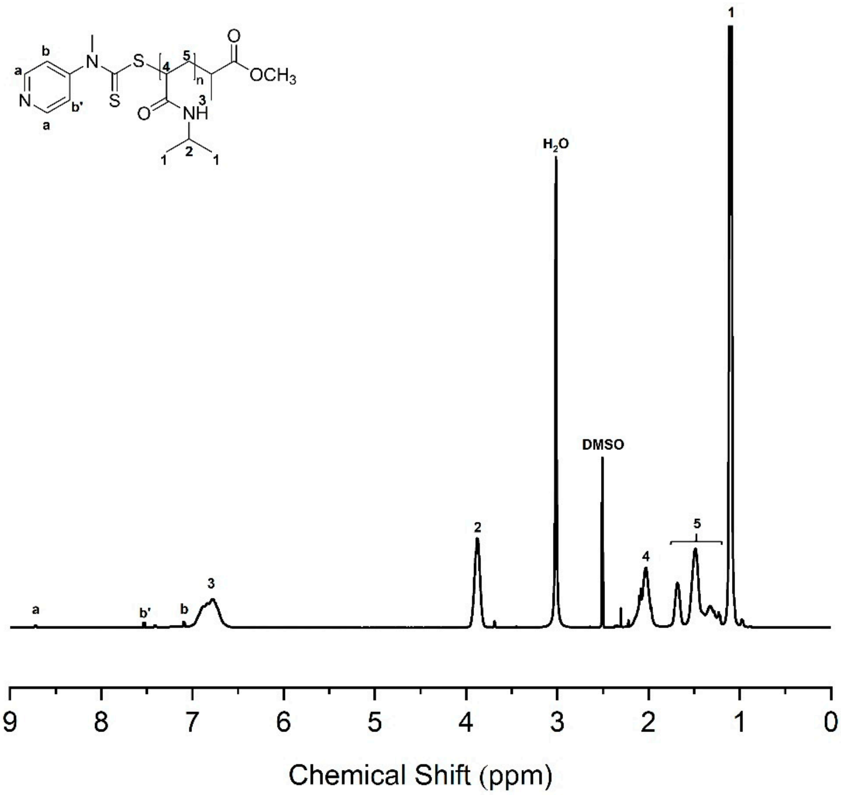

Figure S2. ^{1}H NMR spectrum of PNIPAM$_{258}$ macro-RAFT agent (400 MHz, DMSO-d_6, 100 °C). I ntegration ratio of 1H of **a** : 1H of **2** = 2 : 258.

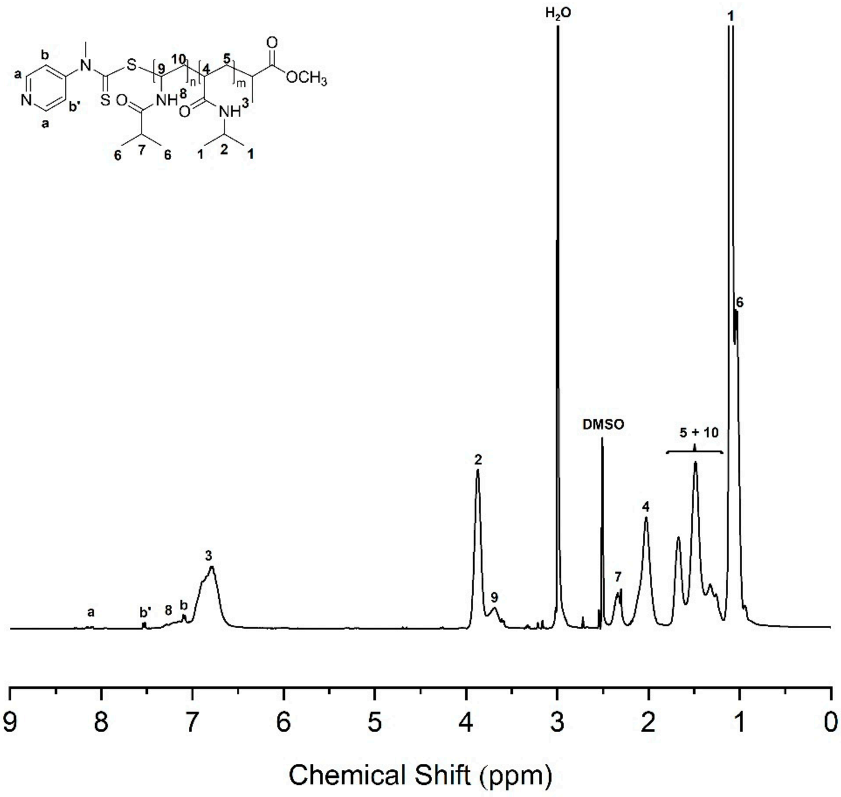

Figure S3. ^{1}H NMR spectrum of PNIPAM$_{258}$-*b*-PNVIBA$_{67}$ (400 MHz, DMSO-*d*$_6$, 100 °C). Integrati on ratio of 1H of **4** : 1H of **7** = 258 : 67.

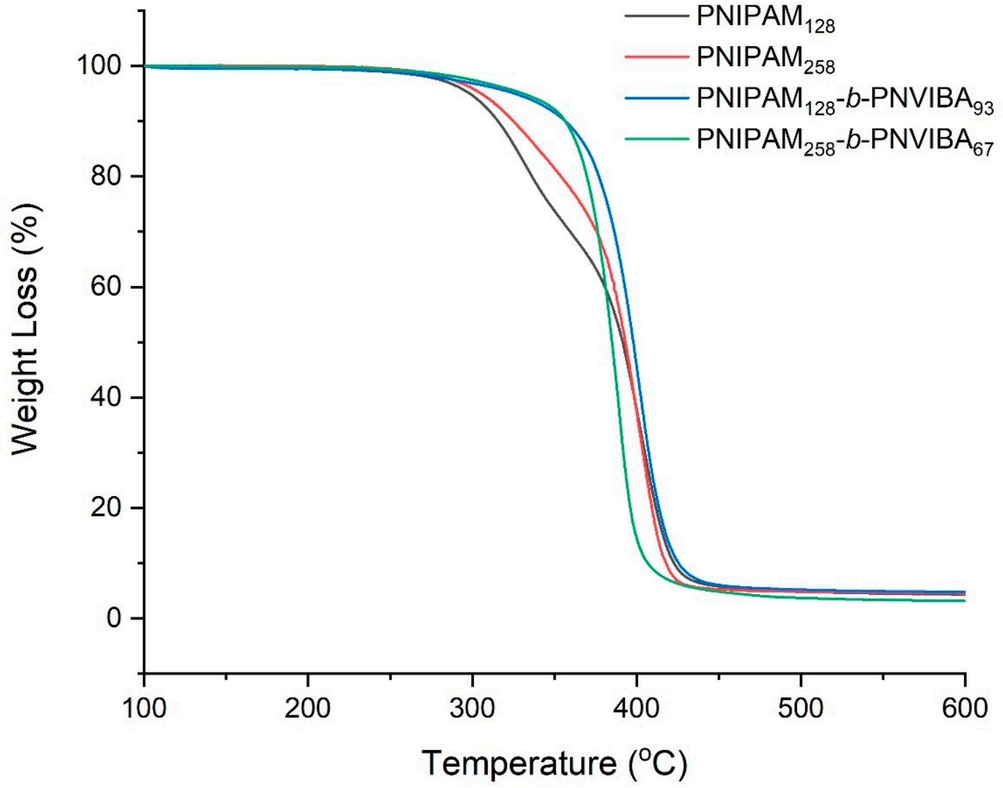

Figure S4. Thermogravimetric Analysis (TGA) of PNIPAM macro-RAFT agent and PNIPAM-b-PNVIBA diblock copolymers. Heating rate : 10 °C/min, temperature range : 100 to 600 °C under N_2 co ndition. 5% decomposition temperature (5% weight loss temperature, T_{d5}) was identified. $PNIPAM_{128}$ macro-RAFT agent T_{d5} = 298.5 °C, $PNIPAM_{258}$ macro-RAFT agent T_{d5} = 316.4 °C, $PNIPAM_{128}$-*b*-$PNVIBA_{93}$ T_{d5} = 325.8 °C, $PNIPAM_{258}$-*b*-$PNVIBA_{67}$ T_{d5} = 330.5 °C.

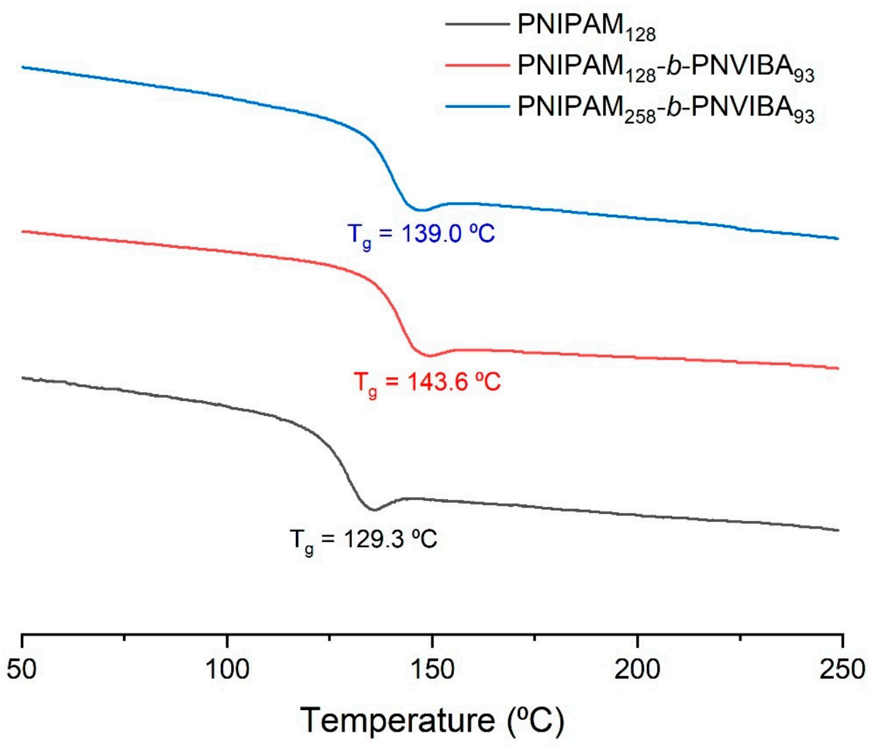

Figure S5. Differential Scanning Calorimetry measurement (DSC) of PNIPAM macro-RAFT agent and PNIPAM-*b*-PNVIBA diblock copolymers. Heating rate : 10 °C/min, and temperature range : -10 to 250 °C under N_2 condition. The data were obtained during the second heating cycle. Glass transition temperature (T_g) of $PNIPAM_{128}$ macro-RAFT agent is 129.3 °C, T_g of $PNIPAM_{128}$-b-$PNVIBA_{93}$ is 143.6 °C, and T_g of $PNIPAM_{128}$-*b*-$PNVIBA_{93}$ is 139.0 °C.

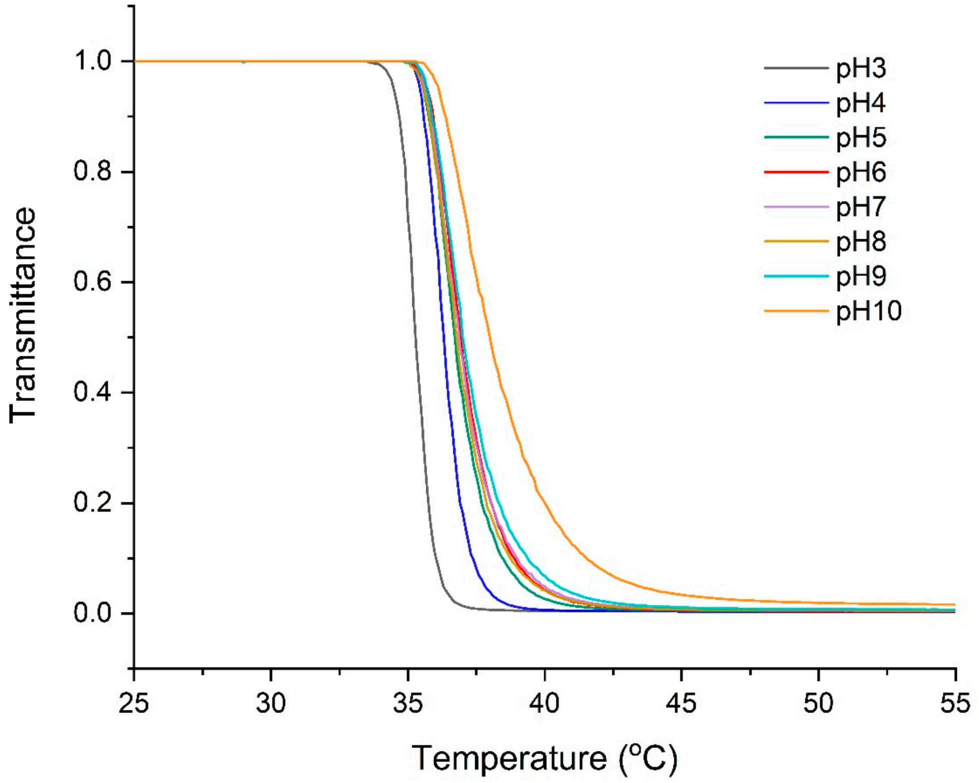

Figure S6. Temperature-dependent change of transmittance to determine thermo-responsive char acter of $PNIPAM_{128}$-*b*-$PNVIBA_{93}$ with changing pH. Range of pH : 3-10. Heating rate: 0.1 °C/m in. Could point (CP) is the temperature when transmittance becomes 0.5. CP of each samples : CP_{pH3} = 36.3 °C, CP_{pH4} = 36.3 °C, CP_{pH5} = 36.7 °C, CP_{pH6} = 36.9 °C, CP_{pH7} = 36.9 °C, CP_{pH8} = 36.9 °C, CP_{pH9} = 37.0 °C, CP_{pH10} = 37.8 °C

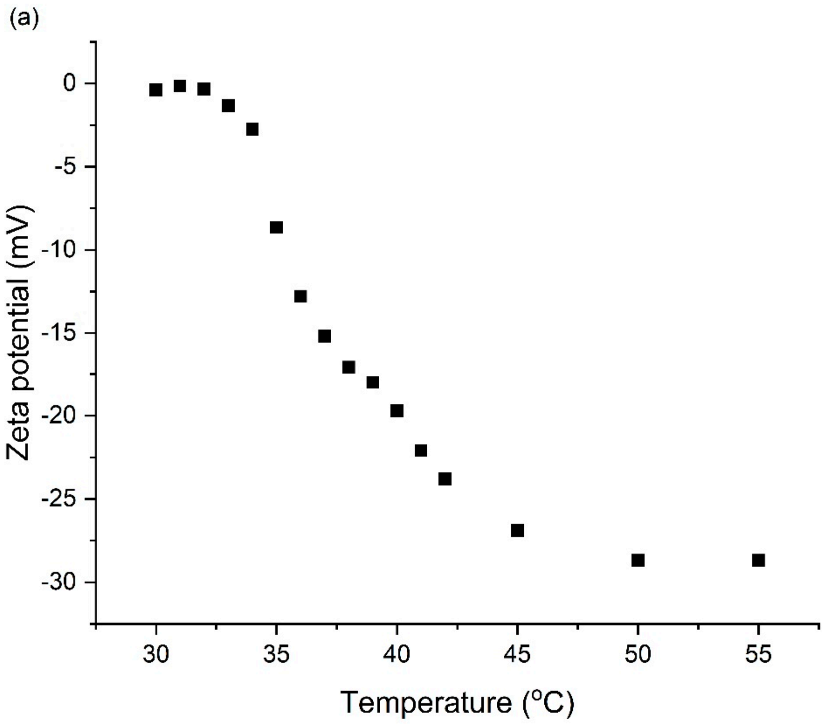

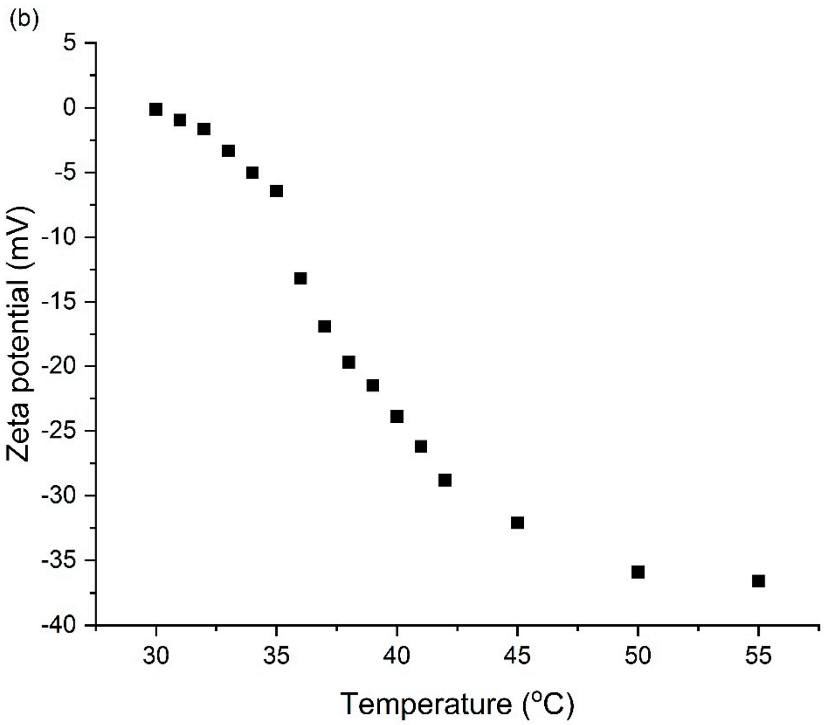

Figure S7. Temperature dependence of the zeta potential for PNIPAM-*b*-PNVIBA polymer soluti ons. (a) $PNIPAM_{128}$-*b*-$PNVIBA_{93}$ solution, (b) $PNIPAM_{258}$-*b*-$PNVIBA_{67}$ solution. Polymer concentra tion : 0.5 mg/mL

Supplementary Tables

Table S1. Variable temperature DLS analysis results of $PNIPAM_{128}$ macor-RAFT agent

Temperature	Eff. Diam. (nm)	Polydispersity	Baseline Index	Count Rate (kcps)	Diffusion Coeff. (cm²/s)
25.0 °C	6.51	0.274	0.0	6.4	7.543E-07
26.0 °C	7.23	0.020	0.0	6.4	6.960E-07
27.0 °C	4.95	0.345	0.0	6.4	1.044E-06
28.0 °C	5.40	0.337	0.0	6.5	9.814E-07
29.0 °C	9.32	0.308	2.8	6.6	5.829E-07
30.0 °C	9.32	0.316	0.0	7.0	5.969E-07
31.0 °C	9.16	0.351	0.0	7.2	6.230E-07
32.0 °C	11.68	0.354	0.0	7.6	5.001E-07
33.0 °C	40.93	0.363	0.0	8.9	1.463E-07
34.0 °C	83.77	0.351	0.0	15.1	7.318E-08
35.0 °C	163.31	0.166	0.0	181.9	3.844E-08
36.0 °C	284.14	0.052	0.0	1,693.7	2.261E-08
37.0 °C	418.45	0.139	0.0	542.9	1.569E-08
38.0 °C	494.92	0.126	3.4	500.0	1.358E-08
39.0 °C	493.14	0.184	9.1	453.3	1.394E-08
40.0 °C	474.28	0.117	9.1	460.6	1.481E-08
41.0 °C	497.88	0.056	9.9	476.8	1.442E-08
42.0 °C	491.51	0.123	8.5	491.0	1.493E-08
43.0 °C	522.80	0.414	8.3	499.3	1.433E-08
44.0 °C	485.26	0.011	6.7	482.9	1.577E-08
45.0 °C	536.37	0.534	9.2	483.5	1.458E-08
46.0 °C	521.76	0.296	9.7	485.2	1.529E-08
47.0 °C	547.19	0.529	9.0	485.6	1.488E-08
48.0 °C	536.54	0.535	7.9	485.4	1.549E-08
49.0 °C	546.36	0.475	8.1	479.5	1.554E-08
50.0 °C	551.34	0.516	9.9	479.7	1.570E-08
51.0 °C	563.87	0.634	9.1	478.3	1.565E-08
52.0 °C	548.57	0.598	7.8	470.1	1.641E-08
53.0 °C	537.65	0.352	8.8	466.6	1.709E-08
54.0 °C	546.51	0.441	9.0	466.0	1.713E-08
55.0 °C	551.79	0.457	9.2	460.9	1.729E-08

Table S2. Variable temperature DLS analysis results of PNIPAM$_{128}$-*b*-PNVIBA$_{93}$ diblock copolymer

Sample ID	Eff. Diam. (nm)	Polydispersity	Baseline Index	Count Rate (kcps)	Diffusion Coeff. (cm²/s)
25.0 °C	18.23	0.328	0.0	9.4	2.760E-07
26.0 °C	22.43	0.355	0.0	9.4	2.243E-07
27.0 °C	26.04	0.351	0.0	9.6	1.985E-07
28.0 °C	26.72	0.319	0.0	9.5	2.187E-07
29.0 °C	24.08	0.339	0.0	9.9	2.255E-07
30.0 °C	26.97	0.337	0.0	9.6	2.382E-07
31.0 °C	26.72	0.319	0.0	9.4	2.187E-07
32.0 °C	21.35	0.353	0.0	9.6	2.871E-07
33.0 °C	25.22	0.357	0.0	9.4	2.374E-07
34.0 °C	32.09	0.337	0.0	10.2	1.956E-07
35.0 °C	44.09	0.325	0.0	10.5	1.262E-07
36.0 °C	53.63	0.337	0.0	12.4	1.038E-07
37.0 °C	160.90	0.121	0.0	133.2	4.081E-08
38.0 °C	294.89	0.122	0.0	944.1	2.280E-08
39.0 °C	343.25	0.111	5.7	596.7	2.003E-08
40.0 °C	378.61	0.186	8.8	613.4	1.855E-08
41.0 °C	379.21	0.115	9.9	540.3	1.893E-08
42.0 °C	353.27	0.142	8.6	561.7	2.078E-08
43.0 °C	321.94	0.143	8.8	527.8	2.328E-08
44.0 °C	292.07	0.176	8.4	522.4	2.621E-08
45.0 °C	277.95	0.058	9.4	507.2	2.813E-08
46.0 °C	266.65	0.034	8.4	507.8	2.992E-08
47.0 °C	257.89	0.008	9.8	506.1	3.157E-08
48.0 °C	251.00	0.037	9.3	490.0	3.312E-08
49.0 °C	245.49	0.056	9.6	490.6	3.457E-08
50.0 °C	244.27	0.094	8.8	472.0	3.543E-08
51.0 °C	233.10	0.055	8.9	470.9	3.787E-08
52.0 °C	234.47	0.079	9.4	468.1	3.840E-08
53.0 °C	235.83	0.102	9.5	471.4	3.896E-08
54.0 °C	236.67	0.151	9.7	472.4	3.955E-08
55.0 °C	236.21	0.182	9.4	473.7	4.038E-08
